# Effect of transcranial direct current stimulation and multicomponent training on functional capacity in older adults: protocol for a randomized, controlled, double-blind clinical trial

**DOI:** 10.1186/s13063-020-4056-2

**Published:** 2020-02-19

**Authors:** Glaucio Carneiro Costa, João Carlos Ferrari Corrêa, Soraia Micaela Silva, Simone Dal Corso, Stefany Ferreira da Cruz, Micaelly de Souza Cunha, Paulo Henrique Leite Souza, Marcella Leiva Saldanha, Fernanda Ishida Corrêa

**Affiliations:** 1 Postgraduate Program in Rehabilitation Sciences, Nove de Julho University (UNINOVE), São Paulo, SP Brazil; 2 Physiotherapy Course, Nove de Julho University (UNINOVE), São Paulo, SP Brazil

**Keywords:** tDCS, Elderly, Multicomponent training, Functional capacity, Activities of daily living

## Abstract

**Introduction:**

When physical activity contains training of at least three components such as balance, coordination and strength, among others, it is called multicomponent training. This type of training is recommended for improving the functional capacity in elderly individuals but has no lasting effects. The association of transcranial direct current stimulation (tDCS) with other types of therapy has been shown to facilitate the enhancement and prolongation of therapy outcomes.

**Aim:**

The objective of this study is to evaluate the effect of multicomponent training associated with active or sham tDCS on the performance of functional capacity in the elderly before treatment, after treatment and 30 days after the end of treatment. The secondary objective will be to correlate the performance of the primary outcome (functional capacity assessed by the Glittre Daily Life Activity Test) with walking capacity (by 6-min walk test), balance (with the mini-Balance Evaluation Systems Test), functional independence (by the Functional Independence Measure) and quality of life (with the World Health Organization Quality of Life Instrument).

**Methods:**

Twenty-eight elderly people from the community will participate in the study, and will be randomized into two groups: 1) multicomponent training associated with active tDCS; and 2) multicomponent training associated with sham tDCS. The multicomponent training sessions will be held twice a week for 12 weeks, totaling 24 sessions. The tDCS will be administered over the dominant dorsolateral prefrontal cortex at the same time as multicomponent training, with an intensity of 2 mA, for 20 min. The evaluations will be made pretraining, after 24 training sessions and 30 days after the end of the training.

**Discussion:**

We hypothesize that tDCS, when associated with multicomponent training, can potentiate and prolong the effects of this training on the functional capacity of the elderly. If this hypothesis is confirmed, this protocol may contribute to a longer-lasting physical rehabilitation of the elderly, encouraging them to maintain their independent daily activities for longer.

**Trial registration:**

The study was registered in the Brazilian Clinical Trial Registry (RBR-2crd42) and received approval from the Human Research Ethics Committee of University Nove de Julho, São Paulo, Brazil (process number 3.077.953).

## Introduction

The World Health Organization (WHO) defines older adults as individuals with a chronological age equal to or more than 60 years in developing countries and 65 years in developed countries [1]. It is estimated that the population of older adults will number approximately 1.2 billion by the year 2025 and will increase to approximately two billion by the year 2050 [1, 2]. In Brazil, the number of older adults is expected to reach approximately 300 million by the year 2050 [3]. Therefore, one of the goals of the Health Ministry in Brazil is to create strategies to address the needs of this growing population so that older adults can age with health, functional independence and quality of life [4].

Functional independence refers to the ability to perform functional activities without the need for other people. This requires the individual to have security to walk, strong muscles, balance, motor coordination, and flexibility. These skills can be enhanced by engaging in daily physical activity through exercise programs, such as multi-component training recommended for seniors by the American College of Sports Medicine [5–12] and involving the training of at least three of these features (or components) in a single session [5–12].

Multicomponent training is effective for improving functional capacity and improving the performance of activities of daily living [13]. However, to observe these effects, prolonged training of 3 to 6 months is required. This time is needed for neurophysiological changes, such as the release of brain-derived neurotrophic factor, to become apparent. However, once this training is stopped, the effects do not last long [14].

Studies with transcranial direct current stimulation (tDCS) [15–21] combined with motor and cognitive therapies have shown that this technique is capable of stimulating neuroplasticity and facilitating motor learning [22], increasing and prolonging the effects of different therapies [23].

tDCS is a neuromodulatory technique that induces excitability of the cerebral cortex without inducing action potential and can modify the resting potential of the neuronal membrane, consequently modulating the neuronal firing rate [23]. It consists of the application of a lowintensity electric current in specific regions properly measured under the scalp using two electrodes that act on the balance of ions inside and outside the neural membrane, stimulating changes in the resting threshold [23]. Anodic current increases cortical excitability, favoring membrane depolarization, and cathodic current decreases cortical excitability, favoring membrane hyperpolarization [20, 21].

Based on this information, this study hypothesizes that tDCS is capable of enhancing and prolonging the effects of multicomponent training on the functional capacity of the elderly, making this type of physical training more effective.

### Objectives

The main objective of this study will be to evaluate the functional capacity of the elderly participants before and after participation in physical activity with multicomponent training associated with active or sham tDCS and to verify if these results are maintained 30 days after the end of the training.

Secondary outcomes will correlate the performance of the primary outcome (functional capacity) with the walking capacity, balance, muscle strength and functional independence after multicomponent training alone or when associated with tDCS.

### Trial design

The study is a randomized, double-blind, placebo-controlled clinical trial. This study will follow the recommendations of the SPIRIT statement, whose checklist can be found in Additional file 1. The study was registered in the Brazilian Clinical Trial Registry (RBR-2crd42) and received approval from the Human Research Ethics Committee of University Nove de Julho, São Paulo, Brazil (process number 3.077.953).

## Methods

To participate in the study, elderly individuals who have the following eligibility criteria will be recruited from the community.

To be included, participants must be between 60 and 80 years old, sign an informed consent form, be able to independently stand and walk without a care device (i.e., be nonfrail elderly) as classified by the Physical Performance Test scale [24] and be hemodynamically stable (blood pressure <140 and 90 mmHg) according to the guidelines of the Brazilian Society of Cardiology [25].

Exclusion criteria include: experiencing amputation or immobilization of a lower limb; musculoskeletal conditions that would restrict the execution of training; chronic or progressive neurological conditions of central or peripheral origin that cause motor limitations; pain that impedes the execution of training and evaluation; diagnosis of acute coronary syndrome or severe heart condition (score >4 on the New York Heart Association functional classification [25]); presence of chronic obstructive pulmonary disease, asthma, cystic fibrosis, or interstitial lung disease; cognitive impairment with a score less than 26 literate and 13 illiterate in the Mini-Mental State Examination [26]; visual impairment that prevents training; pacemaker use; or any contraindication to the use of tDCS, such as history of seizure or recurrent epilepsy, tumor of the brain, metallic material near the stimulation site, skin infection or injury at the electrode application site.

### Intervention

#### Multicomponent training

According to the American College of Sports [10], for physical activity to be considered multicomponent training, it must contain at least three components. Components can be activities based on coordination of movement, balance, flexibility, cardiorespiratory capacity, muscle endurance, agility and cognition.

Therefore, our multicomponent training protocol was based on these recommendations, and will be performed in the following sequence: 1) a warm-up period, during which the individual must walk along a straight and flat track of 20 m for 5 min; 2) a cardiorespiratory capacity component where the individual should ride a stationary exercise bike for 20 min; 3) a lower limb strength training component, where the individual should sit and stand up from a chair without support for the upper limbs for 5 min; 4) a balance component, where the individual should walk 10 m along a path that will contain obstacles to deflect them (zigzag in cones) and another 10 m walk on unstable surfaces (carpets of different thickness) and up and down two steps; the individual will perform these components for 10 min; 5) an upper limb strength training, coordination, balance, and cognition component, where the individual should transfer three balls, weighing 1 kg each, from an upper shelf (at shoulder level) to a lower shelf (at pelvis waist level) and then to the floor, and then return the objects to the top shelf in the same sequence for 10 min; and 6) a flexibility and cooling-down component, where the individual will perform stretching in the standing position and relaxation in the sitting position for 5 min.

The sessions will be individualized, lasting 50 min, and in the 20-min period of training on the bike tDCS will be applied.

The Karvonen formula (220 – age) [27] will be used to determine the maximum heart rate of each participant, considering effort on a submaximum test of 60% to 80% of predicted heart rate. Before initiating training, the exercises will be demonstrated to familiarize the participants with the procedures. The treatment will be performed twice a week for 12 weeks (for a total of 24 sessions).

The following variables will be measured before, during (if necessary) and after each multicomponent training session as a protective measure against possible risks: heart rate, respiratory rate, blood pressure, oxygen saturation and lower limb fatigue level [28].

If the participants have any symptoms or signs that suggest a risk, the training will be stopped immediately. At the treatment site, there is a fire department rescuer who can be called for a medical emergency. All training sessions will be assisted and monitored by an experienced physiotherapist and two physiotherapy students positioned next to the participant.

#### Transcranial direct current stimulation

tDCS will be administered using the DC-STIMULATOR PLUS (NeuroConn) at 2 mA for 20 min during exercise bike training. Two nonmetallic electrodes will be used, wrapped in sponge previously moistened with saline solution. The anode electrode (5 × 5 cm) will be positioned over the left dorsolateral prefrontal cortex (F3) according to the International 10–20 Electroencephalogram System and the cathode electrode (5 × 7 cm) will be positioned supraorbital contralateral to the anode.

For sham tDCS, the electrode placement will be the same as the active protocol, but the stimulator equipment will only be turned on for the initial 30 s, following the parameters set for the stimulated mode device. Thus, participants will have an initial sense of stimulation but will not receive stimulation for the remainder of the 20 min. This is considered a valid form of control in studies with tDCS [29].

#### Determination of potential side effects

Possible adverse effects stemming from noninvasive brain stimulation will be determined using the tDCS Side Effects Questionnaire (translated into Portuguese) [30] after each session and immediately after the intervention.

### Outcomes

The primary outcome of this study will be functional capacity, which will be measured by the Glittre Activities of Daily Living (Glitter-ADL) test [31]. The Glitter-ADL test will be administered on three occasions: 1) pretraining; 2) after 24 screening sessions; and 3) 30 days after the end of training.

Secondary outcomes will be walking capacity measured by the 6-min walk test (6MWT) [32], balance measured by the mini-Balance Evaluation Systems Test (mini-BESTest) [33], functional independence measured by the Functional Independence Measure (FIM) [34], quality of life measured by the Quality of Life Instrument (QOLS) [35] and muscle strength measured by the Medical Research Council scale [36].

**Fig. 1:**
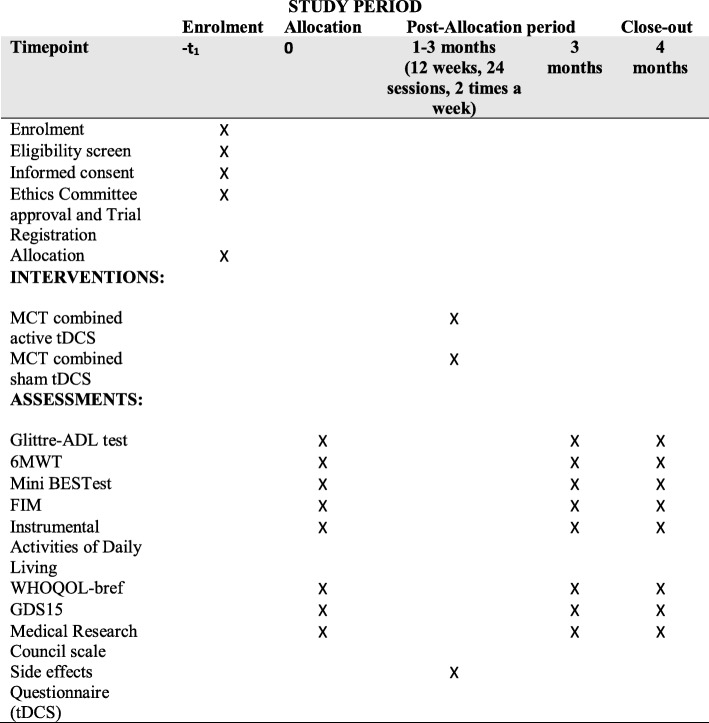
Schedule of enrollment, interventions, and assessments. 6MWT 6-min walk test, BESTest Balance Evaluation Systems Test, FIM Functional Independence Measure, GDS15 15-item Geriatric Depression Scale, Glittre-ADL Glittre Activities of Daily Living, MCT multicomponent training, tDCS Transcranial direct current stimulation, WHOQOL-bref World Health Organization Quality of Life Instrument

The study diagram is represented in Fig. 1.

#### Sample size calculation

A pilot study was conducted with ten older adults separated into two groups (*n* = 5) to determine the number of participants we need to recruit. The sample size was calculated considering the primary outcome (functional capacity evaluated using the Glittre-ADL test), a significance level of α = 0.05 and 80% power, resulting in 22 participants to which 20% was added to compensate for possible dropouts. Thus, the sample will be composed of 28 participants, 14 in the intervention group (multicomponent training combined with active tDCS) and 14 in the control group (multicomponent training combined with sham tDCS). The calculation was performed using the G*POWER 3 software and repeated-measures analysis of variance (ANOVA) F test, considering the difference in the time required to complete the test in the pilot study (mean of 172.05 s in group 1 and 203.82 s in group 2, with a standard deviation of 30.79 s).

### Recruitment

Participants will be recruited from physiotherapy clinics at Nove de Julho University, Sao Paulo, Brazil.

To try and maintain participant retention until the end of the study, a weekly call routine will be created to remind participants and encourage them to continue participating through contact via WhatsApp or telephone calls.

### Allocation and blindness

The NeuroConn DC-STIMULATOR PLUS device has settings that allow one to select the active or sham protocol by codes consisting of a five-digit numeric sequence (provided by the manufacturer). There are 200 different possibilities (100 for each stimulus type of active or sham), so each participant will have their own code. Access to these numbers will only be provided for one of the researchers (researcher 1). This researcher will randomize the participants (http://www.randomization.com) and enter the codes according to the randomization (active group or sham) into the equipment, and thus it is not possible for the researcher who will apply the treatment and evaluate the results (researcher 2) to know the code and what is the treatment, ensuring blinding.

To ensure blinding of the participants and researcher 2 (a double-blind study) during stimulation, the device will appear switched on for both protocols (with no different external visual cues); however, for the active protocol the device will be programmed to operate for 20 min. For the sham protocol, the device will emit current for 20 s and then reduce this to zero (this programming is done by the manufacturer).

Researcher 2 will explain to the participant that they may feel a slight tingling that may continue or subside over time.

When treatment is over, researcher 1 will give researcher 2 a table for data analysis, with the names of participants divided into groups 1 and 2 (according to randomization), without treatment identification.

Researcher 2 will only know which group has received active and which group has received sham tDCS when the 30-day follow-up statistical analyses are complete.

To check blinding to treatment, a questionnaire will be delivered to each participant and researcher 2 at the end of each session asking whether the treatment was applied as active or sham tDCS.

### Data collection methods

#### Evaluations to characterize the elderly participants

##### Fragility assessment

To characterize the elderly regarding fragility we will use the Physical Performance Test scale [24], which classifies the elderly as nonfragile (32 to 36 points), slightly fragile (25 to 31 points) or moderately fragile (17 to 24 points).

This scale scores the performance of nine tasks: 1) raise a book from waist height to a shelf above shoulder level; 2) put on and take off a coat (participants put on and take off an appropriately sized standard apron as quickly as possible); 3) participants retrieve a penny coin that is located in front of the foot as quickly as possible; 4) participants sit, stand fully, and sit back in a chair that has a seat height of 40 cm without using their hands five times as quickly as possible; 5) participants rotate clockwise and counterclockwise quickly, but safely, and are subjectively rated for stability and the ability to produce a rotating motion; 6) walking 15 m by walking 7 m in a straight line, turning, and returning to the initial starting place as quickly as possible and safely; 7) climb 10 flights of stairs one at a time as fast as possible; 8) participants climb four flights of stairs (one point is given for each flight of stairs completed); and 9) participants are scored according to their ability to keep their feet together for 10 s.

The score ranges from 0 to 4 for each task, with a maximum score of 36, being: 0 = impossible to complete; 1 = ends with difficulty, above 10 seconds; 2 = ends with difficulty in 10 seconds; 3 = ends safely over 10 seconds; and 4 = finishes safely in 10 seconds.

This Physical Performance Test scale was adapted into Portuguese by Mitre et al. [24]. The intraclass correlation (ICC) was 0.81 (95% confidence interval (CI) 0.51–0.93) for the reliability of the same examiner, and ICC was 0.86 (95% CI 0.63–0.95) for reliability between different examiners. Cronbach’s α coefficient was 0.89 and 0.92, respectively.

##### Depression scale

The 15-item Geriatric Depression Scale is one of the most widely used assessment tools for assessing depressive symptoms [37]. The questionnaire contains 15 questions that are applied as an interview, with the score sums of each question interpreted as follows: 0 to 5 points, no depression; 6 to 10 points, mild depression, 11 to 15 points, severe depression.

The purpose of this scale is to track the symptoms of depression, requiring medical evaluation for a definitive diagnosis. We will investigate whether the participant’s initial emotional state will affect the outcomes. Participants who have symptoms of depression which persist after the study ends will be referred to a specialist.

All assessments will be performed pretraining, after 24 training sessions and 30 days after the end of 24 sessions (30-day follow-up).

#### Assessment of functional capacity

To assess functional capacity, the Glitter-ADL test [31] will be used at three points (pretraining, post-training and 30 days after the end of training). T's an instrument validated for patients with chronic obstructive pulmonary disease, with acceptable reliability (95% CI −0.20 to −0.54).

The Glittre-ADL test is an instrument validated for patients with chronic obstructive pulmonary disease, with acceptable reliability (95% CI −0.20 to −0.54) [31], which has been previously tested in healthy older adults [38, 39], showing excellent intra-rater (ICC 0.90, 95% CI 0.86–0.93) and inter-rater (ICC 0.91, 95% CI 0.88–0.94) reliability (*P* < 0.001 for both). The intra-rater standard measurement error was 0.03 min and the inter-rater standard measurement error was 0.05 min. The minimal detectable change for intra-rater was 0.40 min and for inter-rater was 0.07 min.

The Glittre-ADL test consists of getting up from a chair with a backpack containing one weight (2.5 kg for women and 5.0 kg for men), walking 5 m on a flat track, going up and down steps measuring 17 cm in height and 27 cm in depth, and walking another 5 m to a set of three shelves, one at the top (at shoulder level), one in the middle (at pelvic waist level) and one at floor level. When reaching the shelves, the participant should transfer three balls, weighing 1 kg each, from the top to the middle and then to the bottom shelf and finally from the bottom shelf to the floor, and then return the balls in the same sequence until they reach the upper shelf. The task should be repeated in the reverse sequence until the participant is sitting again.

The complete test consists of five repetitions of this circuit, which should be performed as quickly as possible. Performance is given by the running time (in minutes) of the five laps with a shorter the time indicating better performance, considering a clinically important improvement between 7 and 40 s [38]. To be considered as preserved functional capacity, the test must be performed in approximately 2 min [39].

At the beginning and end of each repeat of the Glittre-ADL test, all of the following measures will be performed for safety monitoring only and will not be used for performance analysis of the Glittre-ADL test as described by Skumlien et al. [31]: blood pressure (measured with a sphygmomanometer; Incoterm model 29,848), heart rate (measured using a Polar FT1 and FT2 monitor), oxygen saturation (measured with a portable digital oximeter) and dyspnea and lower extremity fatigue (using the dyspnea Borg test) [28].

#### Assessment of walking ability

The ability to walk will be assessed by the 6MWT [32], which will be performed at three time points (pre-training, post-training and 30 days after the end of training) in a flat corridor along a 30-m track with markings at each 405 meter.

Participants should walk as fast as possible, without running, while trying to maintain the same pace for 6 min. The participant will be given verbal encouragement every minute using standardized phrases such as “you’re doing well”, “keep up your work”, “you’re halfway there” and “you only have two minutes left” with no expressions or other signs to speed up the pace. If a participant becomes tired, they may slow down or even stop. In the latter case, the timer will not be stopped and the participant will be encouraged to resume the test as soon as they can until the full 6 min are completed.

The test is evaluated by the number of meters traveled during the 6 min, with more meters traveled indicating better test performance.

Heart rate and oxygen saturation will be measured at the third minute of the test. Heart rate, respiratory rate, blood pressure, oxygen saturation and Borg scales [28] for shortness of breath and lower limb fatigue will be assessed at the beginning and end of the test. The 6MWT is a reliable measure that has been validated for the older adult population, with a test–retest reliability of 0.95 [40].

#### Assessment of balance

We will use the mini-BESTest to assess balance [33], which consists of four domains.

- Domain 1 (anticipatory postural adjustments) evaluates the participant’s ability to move from a sitting to standing position, stand on tiptoes, and stand on one foot (right then left).
- Domain 2 (reactive postural responses) evaluates the participant’s ability to perform a protection reaction (during instability) with a step forward, a protective reaction (during instability) with a backward step, and protection reaction (during instability) with a lateral step (right and left).
- Domain 3 (sensory orientation) evaluates the participant’s ability to stand with their eyes open on a firm surface with feet together, stand with their eyes closed on an unstable surface with feet together, and to stand on a ramp with their eyes closed.
- Domain 4 (gait stability) evaluates the participant’s ability to change gear while walking, to walk with head movements (rotate the head horizontally), to walk and turn on an axis, to overcome obstacles, and a timed up and go test associated with another task (double-task) by performing a countdown.

The score is given by the performance in the execution of each domain, and each sub-item of the domains is scored as follows: 2 points for normal performance; 1 point for moderate performance; and 0 points for poor performance. The score ranges from 0 to 32 points, with a balance deficit being defined as a score below 24 points.

The participants will perform the tests without shoes. If they are unable to perform any movement independently, minor adjustments or assistance may be made, but their score will be lower. Instructions for each test will be provided in advance so that the participants can perform each test to the best of their ability. Up to three attempts will be allowed and the best result will be considered for analysis. The mini-BESTest is a reliable measure that has been validated for the elderly population, with test–retest reliability (ICC) ranging from 0.50 to 0.82 [33].

#### Assessment of functional independence

The FIM will be used to assess functional independence [34] and will be administered to the participants in interview form to quantify the degree of assistance required during activities of daily living.

This measure has 18 categories grouped into six dimensions for the evaluation of functional and cognitive status: self-care (eating, grooming, bathing, dressing upper body, dressing lower body, toileting), sphincter control (bladder management, bowel management), transfers (bed/chair/wheelchair, toilet, bathtub/shower), locomotion (walk/wheelchair, stairs), communication (comprehension, expression) and social cognition (social interaction, problem solving, memory).

The value attributed to each item ranges from 1 to 7 and is interpreted as follows: 7, completely independent (activity is executed without assistance); 6, modified independence (activity requires special device, care and safety); 5, supervised (individual requires control, support from another person, but with no physical contact); 4, assistance with minimal contact (individual performs >75% of the task without assistance); 3, moderate assistance (individual performs >50% of the task without assistance); 2, maximum assistance (individual performs >25% of the task without assistance); 1, total assistance (individual performs less than 25% of the task without assistance).

The total is calculated from the sum of the FIM dimensions and ranges from 18 to 126 points. The total is divided into four subscores: 18 points, complete dependence; 19 to 60 points, modified dependence (assistance on up to 50% of the task); 61 to 103 points, modified independence (assistance on up to 25% of the task); 104 to 126 points, complete independence. The Brazilian version of the FIM has been validated [34], with high test reliability (ICC = 0.91 to 0.98) and inter-observer reliability (ICC = 0.87 to 0.98).

#### Assessment of activities of daily living

To assess the independence of individuals to perform activities of daily living will be used the Lawton and Brody Scale [41], reliable and validated measure for the elderly population. The evaluation is performed through a questionnaire containing questions, whose answers can be categorized as independent (3 points), partially dependent (2 points) or dependent (1 point). If the participants reach the maximum score of 25 points, they will be considered completely independent; 5 to 21 points indicates partial dependence and less than 5 points indicates complete dependence.

#### Assessment of quality of life

We will use the WHO Quality of Life Instrument to assess quality of life [35], which will be administered as an interview.

This instrument consists of 26 items divided into four domains: physical, psychological, social relations and environment. Each item will be rated with a score (from 1 to 5) awarded according to the domain being evaluated: very bad, dissatisfied, nothing or never (1 point); too little, bad, sometimes dissatisfied (2 points); medium, neither bad nor good, neither satisfied nor dissatisfied, more or less often (3 points); very, good, quite, good, satisfied and very often (4 points); completely, very good, very satisfied, extremely, completely, very good and always (5 points).

The score ranges from 26 to 130 points, with higher scores indicating a better quality of life.

##### Evaluation of muscle strength

To assess the strength of the lower limbs, consideration will be given to the quadriceps muscle. The test used will be the Medical Research Council evaluation of muscle strength [36] which is tested manually. The interpretation of the result is as follows: if the subject shows no contraction, the degree of force is zero; grade 1 strength is when there is draft muscle contraction; grade 2 strength is when the individual performs muscle contraction and pro-gravity movement (severity eliminated); grade 3 strength is when the individual can perform muscle contraction (movement) against the force of gravity; grade 4 strength is when the individual can perform slight contraction with the force of gravity; grade 5 strength is when the individual can perform contraction with movement against gravity and moderate resistance.

##### Data analysis

To identify data normality and cases of non-normal distribution, data distribution analyses will be performed by visual analysis of the histograms of each treatment group. Descriptive statistics will be used as measures of central tendency and dispersion, mean and standard deviation will be used for quantitative variables, frequency will be used for categorical variables and the median and the interquartile range will be used for nonparametric variables.

An ANOVA of two factors followed by the Bonferroni test will be used for comparative analysis between groups (active and sham tDCS) along the time points (pre- and post-training).

A *P* value <0.05 will be considered indicative of statistical significance, and Pearson correlation will be used to calculate the relationship between Glittre-ADL performance and the secondary outcomes of the capacity to walk (6MWT), balance (mini-BESTest), FIM, instrumental activities of daily living, quality of life, and evaluation of muscle strength at pretraining, following treatment and at 30-day follow-up.

Individual results will be analyzed later for each outcome. These individual data will be an important complement to identifying the clinical relevance of the results [42].

Clinical characteristics, especially outcome-related characteristics, and functional capacity (Glittre-ADL test) will be compared at baseline to ensure homogeneity of the groups. The significance level of *P* < 0.05 refers to the alpha risk adopted in the ANOVA test. If there is a significant difference between groups, times, or interactions between groups and time, the *P* value of the multiple comparison analysis from the Bonferroni post-hoc test will be adopted.

All analyses will be processed using the SPSS program (IBM SPSS Statistics for Windows, Version 22.0, released in 2013, IBM Corp., Armonk, NY, USA). In case of dropouts and missing data, an intention-to-treat analysis will be performed using the appropriate imputation method according to the missing standard, the data will be analyzed when participants receive no treatment (or are under a control condition) depending on the group assigned, and when outcome measures are available an analysis will be performed as if the individuals had received the treatment (or a control condition).

## Discussion

The present study will aim to compare the effects of multicomponent training associated with active and sham tDCS on the functional capacity in elderly participants after 24 training sessions and 30 days after the end of training.

This objective was based on research showing that the use of tDCS combined with other therapies has promising effects, as the sum of the two combined techniques can improve cortical activity and motor learning. Fujiyama et al. [43] observed improvements in upper limb motor skills in the elderly with isometric strength training when associated with the simultaneous use of tDCS compared with a control group.

The hypothesis for this study is that after tDCS application, neuronal membrane depolarization will occur, which will facilitate any subsequent therapeutic stimulus, as long-term potentiation is released after anodal tDCS. Therefore, this effect will facilitate motor learning and cognitive training over a long period [44, 45].

These results are important because older people lose their cognitive ability over time, thus justifying the decision to stimulate the area of the lateral dorsal prefrontal cortex which is responsible for the performance of executive functions and for the planning and precise control of complex movement sequences.

Metuki et al. [46] and Hecht et al. [47] report that, by stimulating this area, better actions regarding decisions, memory and movement precision of motor tasks can be observed.

### Trial status

At the time of manuscript submission, we were recruiting patients. This study was given the registration protocol RBR-2crd42 on 20 December 2018, updated on 3 July 2019. Recruitment commenced on 21 January 2019 and is expected to be completed on 30 July 2019. The study is expected to be completed by November 2019.

## Supplementary information

**Additional file 1.** SPIRIT 2013 checklist: recommended items to address in a clinical trial protocol and related documents.

**Additional file 2.** Free and Informed Consent Form for Participation in Clinical Research.

**Additional file 3.** Performance And Fragility Measures.

**Additional file 4.** Muscle Strength Rating Scale (MRC-Medical Research Coucil).

**Additional file 5.** Mini-Mental State Examination.

**Additional file 6.** Instrumental scale.

**Additional file 7.** The calculation was performed using the G * POWER 3 software.

## Data Availability

Data sharing does not apply to this article because no datasets were generated or analyzed during the present study. Upon completion of this study, the results will be submitted to a compatible journal, as well as for dissemination in conferences and congresses related to the subject of the study. The final study results will be emailed to each participant. If a participant has questions about the results, they can schedule a meeting with the lead researcher to answer any questions.

## Abbreviations

6MWT: 6-min walk test
FIM: Functional Independence Measure
Glittre-ADL: Glittre Activities of Daily Living
Mini-BESTest: Mini-Balance Evaluation Systems Test
tDCS: Transcranial direct current stimulation
WHO: World Health Organization
