# Supplementary material for: Effect of transcranial direct current stimulation and multicomponent training on functional capacity in older adults: protocol for a randomized, controlled, double-blind clinical trial
Source: Trials. 2020 Feb 19;21:203. doi: 10.1186/s13063-020-4056-2 (PMC7031910; doi:10.1186/s13063-020-4056-2)
Supplement: Supplementary file 2 — Additional file 2. Free and Informed Consent Form for Participation in Clinical Research. [file 13063_2020_4056_MOESM2_ESM.pdf]

Free and Informed Consent Form for Participation in Clinical Research:

Name of participant: ____

Address: ____

Phone for contact: ________________ City: ______________ CEP________

Email: ____

The information contained in this chart was provided by the student, **GLAUCIO CARNEIRO COSTA** (PhD Student in Rehabilitation Science at the University of Nove de Julho - UNINOVE), with the aim of signing a written agreement under which the research volunteer authorizes his participation with full knowledge of the nature of procedures and risks to be submitted, with the ability of free will and without any coercion.

1. Title of the Experimental Work:

Evaluation of the functional capacity (ability to perform day-to-day movements) of elderly individuals, before and after transcranial direct current stimulation (tDCS) (electrical stimulation on the scalp), associated with multi-component training (physical exercises): aerobic resistance, muscle strength training, balance training and agility.

2. Objective:

To evaluate the functional capacity (gait, balance, leg strength, arm movements) of the elderly, after electrical stimulation on the scalp and physical exercises.

3. Rationale:

It is known that physical exercises are beneficial to improve gait functions, balance, arm and leg movements of older people, but these exercises associated with electrical stimulation over the head have not been researched and it is expected that electrical stimulation will potentiate the effects of physical exercise, as it has done in other types of therapy.

4. Procedures of the Experimental Phase:

You should come 27 times for this study, 3 times to be evaluated (before, after all the therapy sessions end and 30 days after the end of the therapies) and 24 times for the treatment. The treatment will be done twice a week for 12

weeks. The time required for treatment will be around 1: 30hs (one and a half hours), 40 minutes of preparation and 50 minutes for the session.

The evaluations that will be carried out will be to evaluate your ability to walk, walk up and down steps, pick up objects on shelves, and answer some questions about your activities. performs on a daily basis as well as their emotional state and quality of life and heart rate (heart beats). The time for these evaluations will be 1: 45hs

The treatment will consist of physical exercises in circuit form, realized individually, with duration of 50 minutes. The exercises will be warm-up (Walk in a flat jogger for 5 minutes), with a 20-meter straight course, exercise bike training for (20 minutes), strength training (sit and get up from a chair for 5 minutes), walk straight for 10 meters with obstacles performing zigzag in cones and 10 meters on irregular surfaces with mats and climb two steps and go down two steps for (10 minutes), exercises with arms (transfer objects from a shelf to another and to the floor for 5 minutes) and relaxation with stretching and relaxation in the sitting position for (5 minutes).

At the moment that mr. exercise on the exercise bicycle will be performed by electrical stimulation on the head, which will be performed by an apparatus composed of two rubber electrodes, which will be placed on the head, covered by a sponge, moistened in saline solution. The appliance may be switched on or off, but you will not know if it is switched on or who will place the appliance, this will be defined by lottery by a third person, who will leave the appliance with the necessary parameters for its use). The intention is to know if the device will have any effect on the physical exercises that you will perform. Physical exercises are already reported by other studies that have positive effects on improving your ability to perform day-to-day tasks, so even if the device is off you will benefit from the exercises.

5. Discomfort or Expected Risks:

During electrical stimulation on the scalp, you may feel a tingling and itching sensation and may get a little red below the electrode. There is no risk of burns, seizures, or other serious symptoms that put you at risk of life. During physical activity you may feel tired legs, some type of respiratory discomfort, imbalance, therefore, the vital signs will be constantly monitored.

6. Protective measures against risks:

The protective measures adopted will be: to cease treatment if the symptoms mentioned above are reported by you, causing you discomfort.

Any signs of discomfort, tiredness or at the request of the person, the physical exercises will be interrupted immediately.

You will always be accompanied by both an experienced physiotherapist and two graduate students in physical therapy. They are experienced professionals in these types of activities, with specialization in the therapies mentioned above.

The evaluations involving questions to the patient will be performed indoors by the researcher, and by a companion if the patient so requests. Avoiding constraints.

7. Information: You will be guaranteed that you will receive answers to any question and / or clarification of any doubt regarding procedures, risks, benefits and other matters related to research. Also, the above-mentioned researchers are committed to providing up-to-date information obtained during the study, although this may affect the individual's willingness to continue participating.

8. Research benefits: It is proven in the literature that multi-component training (various physical exercises) can improve the individual's ability to perform their daily activities, however, studies have shown that combining electric stimulus treatment on the scalp to reaching areas of the brain involved in movement can potentiate the effects of exercise as well as prolong its effects.

9. Withdrawal of Consent: You have the freedom to leave the research at any time, for any reason, without any harm to it.

10. Confidentiality: Researchers ensure the privacy of volunteers regarding the confidential data involved in the research.

11. Forms of Reimbursement of Expenses Due to Participation in Research: The participant is fully aware that no economic value will be charged for participating in the study.

12. Research Location: The study will be conducted at the University Nove de Julho - Memorial Campus, Laboratory of Neurology and Neuromodulation, located at Av. Dr. Adolpho Pinto, 109, Barra Funda - São Paulo - SP.

13. Research Ethics Committee (CEP) is an interdisciplinary and independent collegial body that must exist in institutions that carry out research involving human beings in Brazil, created to defend the interests of research participants

in their integrity and dignity and to contribute to the development of the researches within the ethical standards (Norms and Directives Regulating the Research involving Human beings - Res. CNS nº 466/12). The Ethics Committee is responsible for evaluating and monitoring the research protocols in terms of ethical aspects.

Address of the UNINOVE Ethics Committee: Street. Vergueiro nº 235/249 - 12º andar - Liberdade - São Paulo - SP CEP. 01504-001 Phone: 3385-9010 comitedeetica@uninove.br

Ethics Committee Hours: Monday through Friday - 11:30 a.m. to 1:00 p.m. and 3:30 p.m. to 7:00 p.m.

14. Full name and contact numbers of the researchers: Prof. Dr. Fernanda Ishida Corrêa - (011) 97344-0380; Student - Doctorate Glaucio Carneiro Costa - (011) 98602-3021.

COEP (Research Ethics Committee): (011) 3665-9309 / 9310, Rua Vergueiro nº 235/249 - Liberdade - SP - CEP 01504-001 - 12th floor.

15. Possible intercurrences that may arise during the course of the research may be discussed by the means.

São Paulo, ____________________________________ 20___.

16. Post-Information Consent:

I, ____, after reading and understanding this term of information and consent, I understand that my participation is voluntary, and that I can leave at any time of the study, without prejudice. I confirm that I have received a copy of this consent form, and authorize the execution of the research work and the dissemination of the data obtained in this study in the scientific environment.

____

Signature of Participant / Responsible

(All sheets must be initialed by the research participant)

17. I, ____ (Researcher of the person in charge of this research), certify that:

a) considering that ethics in research implies respect for human dignity and the due protection of participants in scientific research involving human beings;

b) This study has scientific merit and the team of professionals duly mentioned in this term is trained, qualified and competent to perform the procedures described in this term;

____

(Glaucio Carneiro Costa)

Signature of Responsible Researcher
