# Supplementary material for: Effect of transcranial direct current stimulation and multicomponent training on functional capacity in older adults: protocol for a randomized, controlled, double-blind clinical trial
Source: Trials. 2020 Feb 19;21:203. doi: 10.1186/s13063-020-4056-2 (PMC7031910; doi:10.1186/s13063-020-4056-2)
Supplement: Supplementary file 3 — Additional file 3. Performance And Fragility Measures. [file 13063_2020_4056_MOESM3_ESM.docx]

| **PERFORMANCE AND FRAGILITY MEASURES** |
| --- |
| 1. Raise a book from waist height to a shelf above shoulder level |
| 2. Put on and take off a coat. Participants put on and take off an appropriately sized standard apron as soon as possible. |
| 3. Participants get a penny that is located in front of the foot as quickly as possible |
| 4. Participants sit, stand fully, and sit back in a chair that has a seat height of 40 centimeters without using their hands five times as quickly as possible. |
| 5. Participants rotate clockwise and counterclockwise quickly but safely. They are subjectively rated for stability and ability to produce rotating motion. |
| 6. Walk 15 meters. The subjects walk 7 meters in a straight line, turn and return to the initial starting place as quickly as possible safely. |
| 7. A flight of stairs. Climb 10 flights of stairs one at a time as fast as possible. |
| 8. Participants climb four flights of stairs. (One point is given for each flight of stairs completed). |
| 9. Participants are scored according to their ability to keep their feet together for 10 seconds. |
