# Supplementary material for: Effect of transcranial direct current stimulation and multicomponent training on functional capacity in older adults: protocol for a randomized, controlled, double-blind clinical trial
Source: Trials. 2020 Feb 19;21:203. doi: 10.1186/s13063-020-4056-2 (PMC7031910; doi:10.1186/s13063-020-4056-2)
Supplement: Supplementary file 4 — Additional file 4. Muscle Strength Rating Scale (MRC-Medical Research Coucil). [file 13063_2020_4056_MOESM4_ESM.docx]

**Muscle Strength Rating Scale (MRC-Medical Research Coucil).**

Muscle strength grading from Medical research council.

Patient strength is rated

Grade 5: Normal strength against total resistance.

Grade 4: Muscle strength is reduced, but there is muscle contraction against resistance.

Grade 3: The joint can be moved only against gravity and without resistance of the examiner.

Grade 2 There is muscle strength and movement articulate only if the resistance gravity is removed.

Grade 1 Only a motion sketch is seen or felt or fasciculations are observed in the muscle.

Grade 0 No movement is observed.

Medical Research Council. Aids to the examination of the peripheral nevous system, Memorandum no. 45, Her Majesty’s Stationery Office, London, 1981.
