# Supplementary material for: Effect of transcranial direct current stimulation and multicomponent training on functional capacity in older adults: protocol for a randomized, controlled, double-blind clinical trial
Source: Trials. 2020 Feb 19;21:203. doi: 10.1186/s13063-020-4056-2 (PMC7031910; doi:10.1186/s13063-020-4056-2)
Supplement: Supplementary file 5 — Additional file 5. Mini-Mental State Examination. [file 13063_2020_4056_MOESM5_ESM.docx]

**MINI-MENTAL STATE EXAMINATION**

(Foistein, Folstein & McHugh,1.975)

Pacient: ________________________________________________

Date of assessment: ___/ ___/ ___

Reviewer:_______________________

**GUIDANCE**

- Day of the week (1 point) .................................................................. ( )
- Day of the month (1 point)................................................................. ( )
- Month (1 point) .................................................................................. ( )
- Year (1 point) .................................................................................... ( )
- Aproximate time (1 point) ............................................................... ( )
- Specific Location (roo mor sector) (1 point) ...................................... ( )
- Institution (residence, hospital, clinic) (1 point)..... ............................ ( )
- Neighborhood or Nearby Street (1 point) .......................................... ( )
- City (1 point) ..................................................................................... ( )
- State (1 point) ................................................................................... ( )

**MEMORY RETAINED**

- Speak 3 unrelated words. Posteriorly

Ask the patient for the 3 words. Give 1 point

for each correct answer.......................................................................( )

**ATTENTION AND CALCULATIONS**

- (100 - 7) successive, 5 times successively (1 point for each correct calculation) ............................................................................................( )

(alternatively, spell WORLD backwards)

**EVOCATION**

- Ask for the 3 words spoken above

(1 point per word) ...........................................................................( )

**LANGUAGE**

- Name a watch and a pen (2 points) ......................................................( )
- Repeat “neither here nor there nor there” (1 point).......................................................................................................( )
- Command: “take this paper with your right hand fold in half and place it on the floor ”(3 points) ............................................................................( )
- Read and obey: “close your eyes” (1 point) ...........................................( )
- Write a sentence (1 point) ......................................................................( )
- Copy a drawing point..............................................................................( )

**SCORE (___/30)**
