# Supplementary material for: Effect of transcranial direct current stimulation and multicomponent training on functional capacity in older adults: protocol for a randomized, controlled, double-blind clinical trial
Source: Trials. 2020 Feb 19;21:203. doi: 10.1186/s13063-020-4056-2 (PMC7031910; doi:10.1186/s13063-020-4056-2)
Supplement: Supplementary file 6 — Additional file 6. Instrumental scale. [file 13063_2020_4056_MOESM6_ESM.docx]

**Instrumental scale**

| 1. Telephone |  |
| --- | --- |
| Able to see numbers, dial, receive and make calls without help | (3) |
| Able to see, answer phone but need special phone or help | (2) |
| Completely unable to use telephone | (1) |
| 1. Trips |  |
| Able to drive your own car or travel alone by bus or taxi; ; | (3) |
| Able to travel exclusively accompanied ; | (2) |
| Completely unable to travel. . | (1) |
| 1. Purchases |  |
| Able to shop if transportation provided | (3) |
| Able to shop exclusively accompanied | (2) |
| Completely unable to shop. | (1) |
| 1. Meals Preparation |  |
| Able to plan and cook complete meals | (3) |
| Able to prepare small meals but unable to cook complete meals alone; | (2) |
| Completely unable to complete any meal. | (1) |
| 1. Housework |  |
| Able to perform heavy housework (such as mopping the floor | (3) |
| Able to do light housework, but need help with heavy tasks ; | (2) |
| Completely unable to perform any housework | (1) |
| 1. Medications |  |
| Able to take medicine at the right time and right dose; | (3) |
| Able to take medicine but need reminders or someone to prepare them | (2) |
| Completely unable to take medicine alone. | (1) |
| 1. Money |  |
| Able to manage purchase needs, write checks and pay bills; | (3) |
| Able to manage buying needs, but needs help with checks and paying bills; | (2) |
| Completely unable to manage money. | (1) |
