# Supplementary figures and images for: Effect of transcranial direct current stimulation and multicomponent training on functional capacity in older adults: protocol for a randomized, controlled, double-blind clinical trial
Source: Trials. 2020 Feb 19;21:203. doi: 10.1186/s13063-020-4056-2 (PMC7031910; doi:10.1186/s13063-020-4056-2)

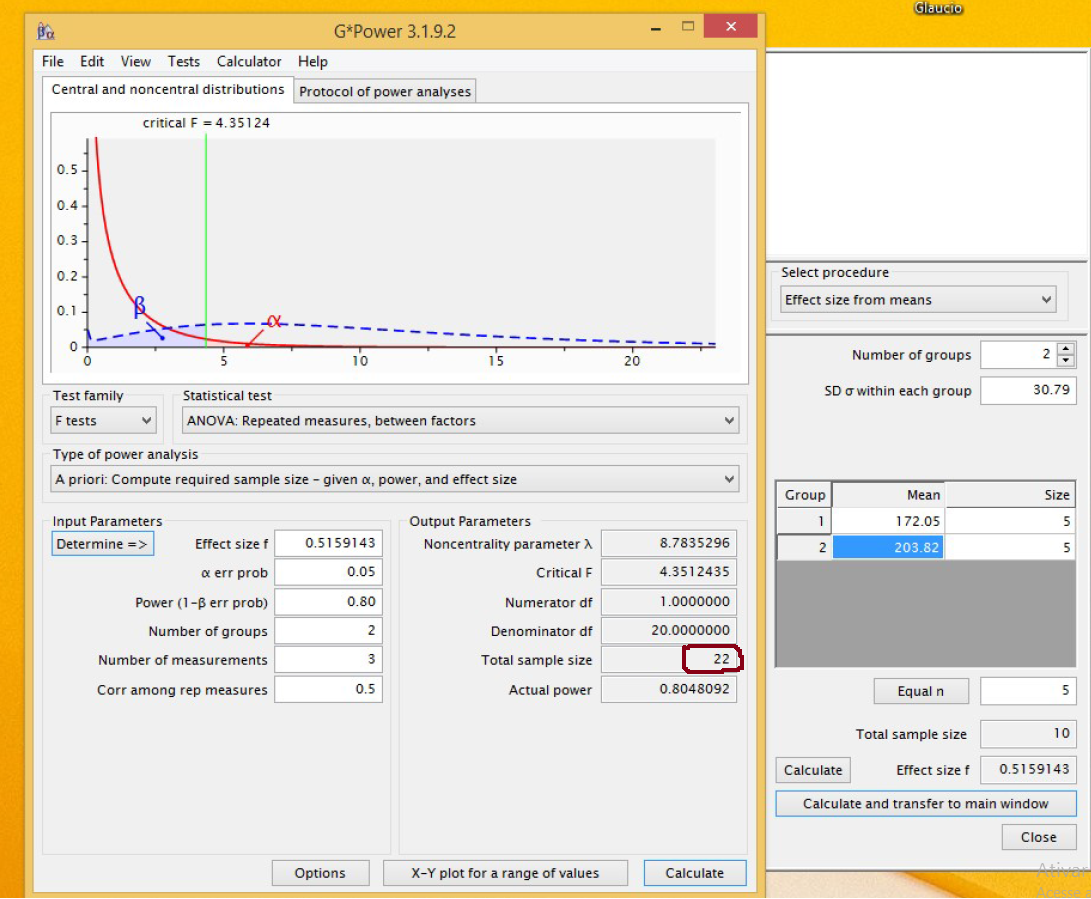

Supplement: Supplementary file 7 — Additional file 7. The calculation was performed using the G * POWER 3 software. [file 13063_2020_4056_MOESM7_ESM.doc]
